# Analysis of the Relation between Periodontitis and Chronic Gastritis/Peptic Ulcer: A Cross-Sectional Study Using KoGES HEXA Data

**DOI:** 10.3390/ijerph17124387

**Published:** 2020-06-18

**Authors:** Soo Hwan Byun, Chanyang Min, Seok Jin Hong, Hyo Geun Choi, Dong Hee Koh

**Affiliations:** 1 Department of Oral & Maxillofacial Surgery, Dentistry, Sacred Heart Hospital, Hallym University College of Medicine, Anyang 14068, Korea; purheit@daum.net; 2 Research Center of Clinical Dentistry, Hallym University Clinical Dentistry Graduate School, Chuncheon 24252, Korea; enthsj@hanmail.net; 3 Hallym Data Science Laboratory, Hallym University College of Medicine, Anyang 14068, Korea; joicemin@naver.com; 4 Department of Otorhinolaryngology-Head & Neck Surgery, Dongtan Sacred Heart Hospital, Hallym University College of Medicine, Hwaseong 18450, Korea; 5 Department of Otorhinolaryngology-Head & Neck Surgery, Sacred Heart Hospital, Hallym University College of Medicine, Anyang 14068, Korea; 6 Department of Internal Medicine, Dongtan Sacred Heart Hospital, Hallym University College of Medicine, Hwaseong 18450, Korea

**Keywords:** periodontitis, gastritis, peptic ulcer, KoGES, *Helicobacter pylori*

## Abstract

The aim of the present study was to investigate the association between periodontitis and chronic gastritis/peptic ulcer using a cross-sectional study design. The present prospective cohort study used epidemiological data from the Korean Genome and Epidemiology Study (KoGES) recorded from 2004 to 2016. Among 173,209 participants, 9983 with periodontitis and 125,336 with no periodontitis were selected. Histories of chronic gastritis and peptic ulcer between periodontitis and no periodontitis participants were analyzed. The participants were questioned around any history of hypertension, diabetes mellitus, hyperlipidemia, cerebral stroke, ischemic heart disease, periodontitis, body mass index, smoking, alcohol consumption, nutritional intake, and financial income. Chi-square tests, independent t-tests, two-tailed analyses were used in statistical analysis of the data. The adjusted odds ratio of chronic gastritis was 2.22 (95% confidence interval [CI] = 2.10–2.34, *p* < 0.001) and that of peptic ulcer was 1.86 (95% CI = 1.74–1.98, *p* < 0.001) in model 2. This study demonstrated that periodontitis was associated with an increased risk of chronic gastritis/peptic ulcer. These findings provide additional evidence for an association between periodontitis and chronic gastritis/peptic ulcer.

## 1. Introduction

Chronic gastritis and peptic ulcer are acid-induced diseases that affect the stomach and proximal duodenum. These diseases are identified by denuded mucosa, and confirmed histologically by extension into the submucosa or muscularis propria [1]. An excessively acidic environment in combination with dietary or stress factors have been considered to induce chronic gastritis/peptic ulcer. However, the discovery of *H. pylori* (*Helicobacter pylori*) infection and the use of non-steroidal anti-inflammatory drugs (NSAIDs) have changed previous perceptions [2].

The prevalence of peptic ulcer is approximately 5 to 10%; recent studies have reported a decrease in prevalence and mortality in the high-income countries [2,3,4]. This phenomenon is most likely related to improved hygiene and a decline in *H. pylori* infections. The decrease in complications of chronic gastritis/peptic ulcer might also be associated with the widespread use of antisecretory medication and stricter use of NSAIDs [3,5,6]. Almost half of the global population is infected by *H. pylori* [7].

The pathophysiology of *H. pylori* infection is not completely understood. Inflammation related to *H. pylori* infection is thought to possibly induce hypochlorhydria or hyperchlorhydria, and this mechanism could be modulated by cytokines [8]. *H. pylori* can activate the H+/K+ ATPase α-subunit, stimulate calcitonin gene-related peptide (CGRP) sensory neurons linked to somatostatin, or inhibit the production of gastrin [9]. Although the occurrence of gastric ulcers is associated with hyposecretion, 10 to 15% of patients with *H. pylori* infection present with hypersecretion [10]. This activates the secretion of histamine, and the eradication of *H. pylori* leads to a decrease in gastrin mRNA expression and an increase in somatostatin mRNA expression [11]. Other pathogenetic factors could possibly include ischemia, metabolic disturbances, systemic disease, viruses, histamine, radiotherapy, eosinophilic, and basophilia infiltration [12].

Periodontal disease involves inflammation and destruction of periodontal tissue, including alveolar bone, periodontal ligament, and gingival tissue by oral bacteria. Periodontal disease is the sixth most prevalent human disease [13]. Epidemiological studies have reported that the prevalence of severe periodontitis ranges from 1% among younger individuals to 39% among individuals over 65 years of age [14,15]. The reported prevalence of periodontitis differs according to the study design and method, and it also varies markedly between countries [14,16]. Between 5% and 25% of the general population present with severe periodontitis, while moderate forms have been found in up to 60% of the general population [14,16]. The total cost of preventive and periodontal dental care in the United States (US) was calculated at $14.3 billion in 1999, with approximately $4.4 billion attributed to periodontal treatment [17]. 

Previous studies have suggested that an association between periodontal disease and systemic diseases, including pulmonary disease, cardiovascular disease, and diabetes mellitus exits [15,18,19]. However, another study has reported that there is still no definitive evidence that treating oral disease has any clinically meaningful effect on the prevention, treatment, or outcomes of any systemic disease [20]. Associations between periodontal disease and systemic diseases are explained by inflammation or the immune response to periodontal pathogens. Periodontitis has characteristics of a pathological change due to dysbiosis within the oral microbiome [21]. The bacterial infection could affect the oral microbiome, and the complex system of the oral microbiome could, in turn, influence systemic inflammatory diseases [22]. An association between periodontal disease and chronic gastritis/peptic ulcer has been investigated in only a limited number of studies [23,24,25]. Yu H et al. demonstrated a significantly positive association between peptic ulcer and periodontal disease in Taiwan [25]. Boylan et al. reported that periodontal disease was linked to an increased risk of gastric and duodenal ulcer [24], while Umeda et al. suggested that patients with periodontitis who harbor *H. pylori* in the oral cavity should be closely monitored [23]. However, these previous studies did not adjust for various confounding factors including hypertension, diabetes mellitus, hyperlipidemia, cerebral stroke, ischemic heart disease, obesity, financial income, smoking, alcohol consumption, nutritional intake, chronic gastritis, and peptic ulcer.

The aim of this study was to investigate the association between periodontitis and chronic gastritis/peptic ulcer using a cross-sectional study design and the KoGES HEXA (Korean Genome and Epidemiology Study Health Examinee) data.

## 2. Materials and Methods

### 2.1. Study Population and Data Collection

The ethics committee of Hallym University (2019-02-020) approved the use of the KoGES HEXA data. KoGES was undertaken between 2004 and 2016. A detailed description of this data was provided in a previous study [26]. As mentioned, among the KoGES Consortium, the HEXA data were singled out for use in the present prospective cohort study. This data was derived from urban residents ≥40 years of age. It consisted of baseline data recorded from 2004–2013, and follow-up data from 2012–2016. A cross-sectional study using data from the prospective cohort study was conducted.

The requirement for written informed consent was waived by the Institutional Review Board.

### 2.2. Participant Selection

**Figure 1:**
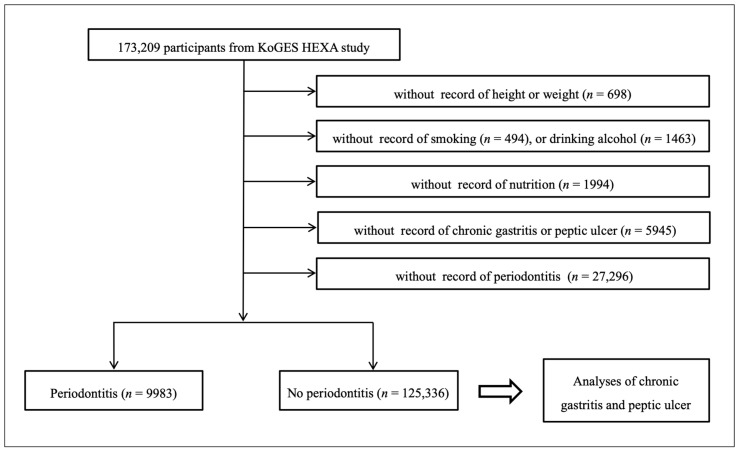
A schematic illustration of the participant selection process. Of a total of 173,209 participants, 9983 participants with periodontitis and 125,336 with no periodontitis were selected.

From a total of 173,209 participants, those who lacked information concerning height or weight (*n* = 698), smoking history (*n* = 494), alcohol consumption (*n* = 1463), nutrition (*n* = 1994), experience of chronic gastritis or peptic ulcer (*n* = 5945), and periodontal status (*n* = 27,296) were excluded. Many participants were excluded as a history of chronic gastritis/peptic ulcer and periodontitis were not surveyed in 2004 and from 2004–2006, respectively. Finally, 9983 participants with periodontitis, and 125,336 without periodontitis (no periodontitis group) were selected (Figure 1). Following selection, the participants’ histories of chronic gastritis and peptic ulcer were analyzed. 

### 2.3. Survey

The participants were asked about any history of periodontitis, chronic gastritis, and peptic ulcer by trained interviewers. The questions were structured as follows “Have you ever had a diagnosis of periodontitis?”; “Do you have any history of chronic gastritis?”; and “Do you have any history of peptic ulcer?”. All questions required yes or no answers and responses were confirmed by dentists and medical doctors BMI (body mass index) was calculated by dividing the participant’s weight in kilograms by height in meters squared (kg/m^2^), using the health checkup data. Smoking histories were categorized as non-smoker (<100 cigarettes over the lifetime), past smoker (quit for longer than one preceding year), and current smoker. Likewise, histories of alcohol consumption were categorized as non-drinker, past drinker, and current drinker. Nutritional intake (total calories (kcal/day), protein (g/day), fat (g/day), and carbohydrate (g/day)) was surveyed using a food-frequency questionnaire, validated by a previous study [27]. Income groups were classed as no-response, low income (<~$2000), middle income (~$2000–$3999), and high income (~≥$4000) relative to monthly household income. 

### 2.4. Statistical Analyses

Chi-square tests were used to compare the relationships between sex, income group, smoking, alcohol consumption, chronic gastritis, and peptic ulcer, while independent *t*-tests were used to compare age, BMI, and nutritional intake.

To analyze the OR (odds ratio) of chronic gastritis and peptic ulcer for periodontitis, crude, model 1 (adjusted for age, sex, BMI, smoking, alcohol consumption, and nutritional intake), and model 2 (model 1 plus chronic gastritis/peptic ulcer) were calculated. 

In the subgroup analyses according to age, the median age (<53 years, and ≥53 years old) was selected as the dividing point. 

Two-tailed analyses were conducted, and P values less than 0.05 were considered significant. 

The data were statistically analyzed using SPSS v. 24.0 (IBM, Armonk, NY, USA).

## 3. Results

The general characteristics of the participants differed between the periodontitis and no periodontitis groups (Table 1). 

The AOR (adjusted ORs) of chronic gastritis was calculated as 2.22 (95% CI (confidence interval) = 2.10–2.34, *p* < 0.001), and that of peptic ulcer 1.86 (95% CI = 1.74–1.98, *p* < 0.001) in model 2 (Table 2). 

In the subgroup analyses according to age and sex, the results were consistent (Table 3). The AOR of chronic gastritis was 1.97 (95% CI = 1.66–2.33) < 53 years of age for men; 2.24 (95% CI = 2.02–2.48) < 53 years of age for women; 2.12 (95% CI = 1.89–2.38) ≥ 53 years of age for men; 2.28 (95% CI = 2.10–2.48) ≥ 53 years of age for women. The AOR of peptic ulcer was 1.67 (95% CI = 1.41–1.99) < 53 years of age for men; 1.88 (95% CI = 1.64–2.16) < 53 years of age for women; 1.93 (95% CI = 1.71–2.17) ≥ 53 years of age for men; 1.84 (95% CI = 1.64–2.05) ≥ 53 years of age for women. 

## 4. Discussion

Despite the reliability of previous studies, the clinical association between periodontitis and chronic gastritis/peptic ulcer has not been evaluated in detail [14,28,29]. For these reasons, the present study was conceived to determine whether periodontitis significantly influenced the likelihood of a diagnosis or severity of chronic gastritis/peptic ulcer. This study demonstrated a significant association between periodontitis and chronic gastritis/peptic ulcer in all ages and sex groups when using the KoGES HEXA data. This result corresponded with those reported by previous studies. 

The association between periodontitis and gastric ulcer can be divided into two types according to *H. pylori*-related and non-*H. pylori*-related situations. The association between periodontitis and *H. pylori*-related chronic gastritis/peptic ulcer can be explained as a gastric infection arising from the intraoral area. However, the transmission pathway of *H. pylori* is not completely understood. It is possible that the intraoral area provides a pathway of infection of *H. pylori* to the stomach and duodenum, and, for example, during gastroscopy, the gastroscope could contact and damage the gastric or duodenal mucosa increasing the risk of *H. pylori* transmission from the intraoral area to the rest of the digestive tract [23]. In this case, the intraoral area would act as the reservoir of *H. pylori*. Therefore, it follows that elimination of *H. pylori* from the oral cavity would be essential to inhibit transmission to the other digestive organs. Previous studies have demonstrated that *H. pylori* in dental plaque might be a possible risk factor for gastric infection [29,30,31,32], while others have investigated the effects of periodontal treatment on *H. pylori* infection in the intraoral and gastric areas [28,33]. Individuals who received treatment for *H. pylori* infection and periodontitis presented with increased rates of *H. pylori* eradication when compared to those who received treatment for *H. pylori* alone [31,33]. A meta-analysis also proposed that periodontal treatment could improve the outcomes of *H. pylori* treatment [28].

The association between periodontitis and gastritis/peptic ulcer may also be explained by a mechanism unrelated to *H. pylori* infection. It is likely that periodontitis is related to systemic inflammation [34]. Some studies have identified an increased level of plasma C-reactive protein in persons with periodontal disease [35,36,37], while others have linked periodontal disease with chronic inflammation, such as cardiovascular disease and cancers of the kidney, pancreas, and lung [18,38,39]. Based on these studies, it appears that periodontal disease may be associated with a systemic inflammation that predisposes an individual to peptic ulceration [24]. 

The major strength of the present study was the large sample size. The HEXA data identified a large number of participants with periodontal disease and a chronic gastritis/peptic ulcer diagnosis. In addition, potential confounders, particularly hypertension, diabetes mellitus, hyperlipidemia, cerebral stroke, ischemic heart disease, BMI, financial income, smoking, alcohol consumption, nutritional intake, chronic gastritis, and peptic ulcer were adjusted to reduce the surveillance bias (Table 1). This minimized the influence from systemic factors. Similarly, Model 2, in Table 2, was designed to exclude the mutual influence of chronic gastritis and peptic ulcer. 

There are a few limitations of the present study. First, information was extracted from self-reported questionnaires. Therefore, the data could be inaccurate. Second, there are various types of periodontitis, and the severity and extent of periodontitis could be different in participants. Hence the term “periodontitis” could be too simplistic for one single pathology definition. Third, the population of this study comprised predominantly Asian and Korean participants, which may limit the generalizability of the findings to other nationalities [24]. Fourth, confounding factors for periodontitis and chronic gastritis/peptic ulcer could be residual. Fifth, the influence of aspirin and NSAID use was not ruled out. Since aspirin and NSAIDs are readily available without a prescription, it was impossible to check the exact frequency and magnitude of use by participants. A pathophysiological association between periodontitis and gastritis/peptic ulcer, in the presence or absence of an *H. pylori* infection, could not be confirmed by this study. Future research into the pathophysiological relationship between the two conditions is required [20]. 

## 5. Conclusions

In conclusion, this study demonstrated that periodontitis was associated with an increased risk of chronic gastritis/peptic ulcer. These findings provide additional support for an association between chronic inflammation, dissemination of *H. pylori*, and ulceration of the digestive tract. Further research is needed with respect to the factors that reduce the risk of periodontitis and chronic gastritis/peptic ulcer.

## Figures and Tables

**Table 1:** General characteristics of participants.

Characteristics	Total Participants		*p*-Value
	Periodontitis	No Periodontitis	
Age (mean, SD, y)	54.8 (7.9)	53.0 (8.3)	<0.001 *
Sex (*n*, %)			<0.001 *
Men	3852 (38.6)	43,410 (34.6)	
Women	6131 (61.4)	81,926 (65.4)	
BMI (mean, SD, kg/m^2^)	24.0 (2.9)	23.9 (2.9)	<0.001 *
Income (*n*, %)			<0.001 *
Missing, no response	766 (7.7)	10,856 (8.7)	
Lowest	3441 (34.5)	35,593 (28.4)	
Middle	3679 (36.9)	49,428 (39.4)	
Highest	2097 (21.0)	29,459 (23.5)	
Smoking status (*n*, %)			<0.001 *
Nonsmoker	6697 (67.1)	91,133 (72.7)	
Past smoker	1796 (18.0)	18,593 (14.8)	
Current smoker	1490 (14.9)	15,610 (12.5)	
Alcohol consumption (*n*, %)			<0.001 *
Non drinker	4792 (48.0)	64,045 (51.1)	
Past drinker	479 (4.8)	4536 (3.6)	
Current drinker	4712 (47.2)	56,755 (45.3)	
Nutritional intake (mean, SD)			
Total calories (kcal/d)	1760.1 (580.0)	1749.4 (569.3)	0.071
Protein (g/d)	58.9 (26.6)	59.8 (26.4)	0.002 *
Fat (g/d)	27.5 (18.5)	28.3 (18.2)	<0.001 *
Carbohydrate (g/d)	315.0 (95.2)	309.8 (92.8)	<0.001 *
Chronic gastritis	2062 (20.7)	12,393 (9.9)	<0.001 *
Peptic ulcer	1262 (12.6)	7724 (6.2)	<0.001 *

d: day; * Independent *t*-test or Chi-square test. Significant at *p* < 0.05.

**Table 2:** Crude and adjusted odds ratios (95% confidence interval) of chronic gastritis and peptic ulcer for periodontitis.

Characteristics	Odds Ratios for Periodontitis					
	Crude ^†^	*p*-Value	Model 1 ^†^	*p*-Value	Model 2 ^‡^	*p*-Value
Total participants (*n* = 134,855)							
Chronic gastritis	2.37 (2.25–2.50)	<0.001 *	2.35 (2.23–2.48)	<0.001 *	2.22 ^a^ (2.10–2.34)	<0.001 *
Peptic ulcer	2.20 (2.07–2.35)	<0.001 *	2.08 (1.95–2.22)	<0.001 *	1.86 ^b^ (1.74–1.98)	<0.001 *

* Logistic regression model, Significant at *p* < 0.05; ^†^ Model 1 was adjusted for age, sex, BMI, smoking, alcohol consumption, and nutritional intake; ^‡^ Model 2 was adjusted as for Model 1 plus peptic ulcer ^a^ or chronic gastritis ^b^ to control for the mutual influence of chronic gastritis and peptic ulcer, respectively.

**Table 3:** Crude and adjusted odds ratios (95% confidence interval) of chronic gastritis and peptic ulcer for periodontitis according to age and sex.

Characteristics	Odds Ratios for Periodontitis					
	Crude ^†^	*p*-Value	Model 1 ^†^	*p*-Value	Model 2 ^‡^	*p*-Value
Age < 53 years old, men (*n* = 21,513)							
Chronic gastritis	2.16 (1.83–2.56)	<0.001 *	2.10 (1.77–2.48)	<0.001 *	1.97 ^a^ (1.66–2.33)	<0.001 *
Peptic ulcer	1.92 (1.62–2.28)	<0.001 *	1.82 (1.54–2.16)	<0.001 *	1.67 ^b^ (1.41–1.99)	<0.001 *
Age < 53 years old, women (*n* = 45,469)							
Chronic gastritis	2.44 (2.21–2.70)	<0.001 *	2.35 (2.13–2.61)	<0.001 *	2.24 ^a^ (2.02–2.48)	<0.001 *
Peptic ulcer	2.34 (1.95–2.56)	<0.001 *	2.13 (1.85–2.44)	<0.001 *	1.88 ^b^ (1.64–2.16)	<0.001 *
Age ≥ 53 years old, men (*n* = 25,749)							
Chronic gastritis	2.28 (2.03–2.55)	<0.001 *	2.29 (2.05–2.57)	<0.001 *	2.12 ^a^ (1.89–2.38)	<0.001 *
Peptic ulcer	2.17 (1.94–2.44)	<0.001 *	2.12 (1.89–2.38)	<0.001 *	1.93 ^b^ (1.71–2.17)	<0.001 *
Age ≥ 53 years old, women (*n* = 42,588)							
Chronic gastritis	2.38 (2.20–2.59)	<0.001 *	2.40 (2.21–2.60)	<0.001 *	2.28 ^a^ (2.10–2.48)	<0.001 *
Peptic ulcer	2.09 (1.88–2.33)	<0.001 *	2.07 (1.86–2.31)	<0.001 *	1.84 ^b^ (1.64–2.05)	<0.001 *

* Logistic regression model, Significant at *p* < 0.05; ^†^ Model 1 was adjusted for age, sex, BMI, smoking, alcohol consumption, and nutritional intake; ^‡^ Model 2 was adjusted as for model 1 plus peptic ulcer ^a^ or chronic gastritis ^b^ to control for the mutual influence of chronic gastritis and peptic ulcer, respectively.
